# Supplementary material for: An iPSC-based in vitro model recapitulates human thymic epithelial development and multi-lineage specification
Source: Nat Commun. 2025 Aug 25;16:7680. doi: 10.1038/s41467-025-62523-1 (PMC12378236; doi:10.1038/s41467-025-62523-1)
Supplement: Supplementary file 1 — Supplementary Information [file 41467_2025_62523_MOESM1_ESM.pdf]

Supplementary Information

An iPSC-based *in vitro* model recapitulates human thymic epithelial development and multi-lineage specification

Yann Pretemer[1], Yuxian Gao[1,2], Kaho Kanai[1,3], Takuya Yamamoto[1,4,5], Kohei Kometani[1], Manami Ozaki[1,3], Karin Nishigishi[1,3], Tadashi Ikeda[6], Huaigeng Xu[1,7], Akitsu Hotta[1], Yoko Hamazaki[1,2]*

[1]Center for iPS Cell Research and Application, Kyoto University, Kyoto, Japan
[2]Laboratory of Immunobiology, Graduate School of Medicine, Kyoto University, Kyoto, Japan
[3]Department of Human Health Sciences, Graduate School of Medicine, Kyoto University, Kyoto, Japan
[4]Institute for the Advanced Study of Human Biology, Kyoto University, Kyoto, Japan
[5]RIKEN Center for Advanced Intelligence Project, Kyoto, Japan
[6]Department of Cardiovascular Surgery, Graduate School of Medicine, Kyoto University, Kyoto, Japan
[7]Eli and Edythe Broad Center of Regeneration Medicine and Stem Cell Research, University of California, San Francisco, San Francisco, CA, USA

*Correspondence: yoko.hamazaki@cira.kyoto-u.ac.jp

This PDF file includes:

Supplementary Figures 1-13

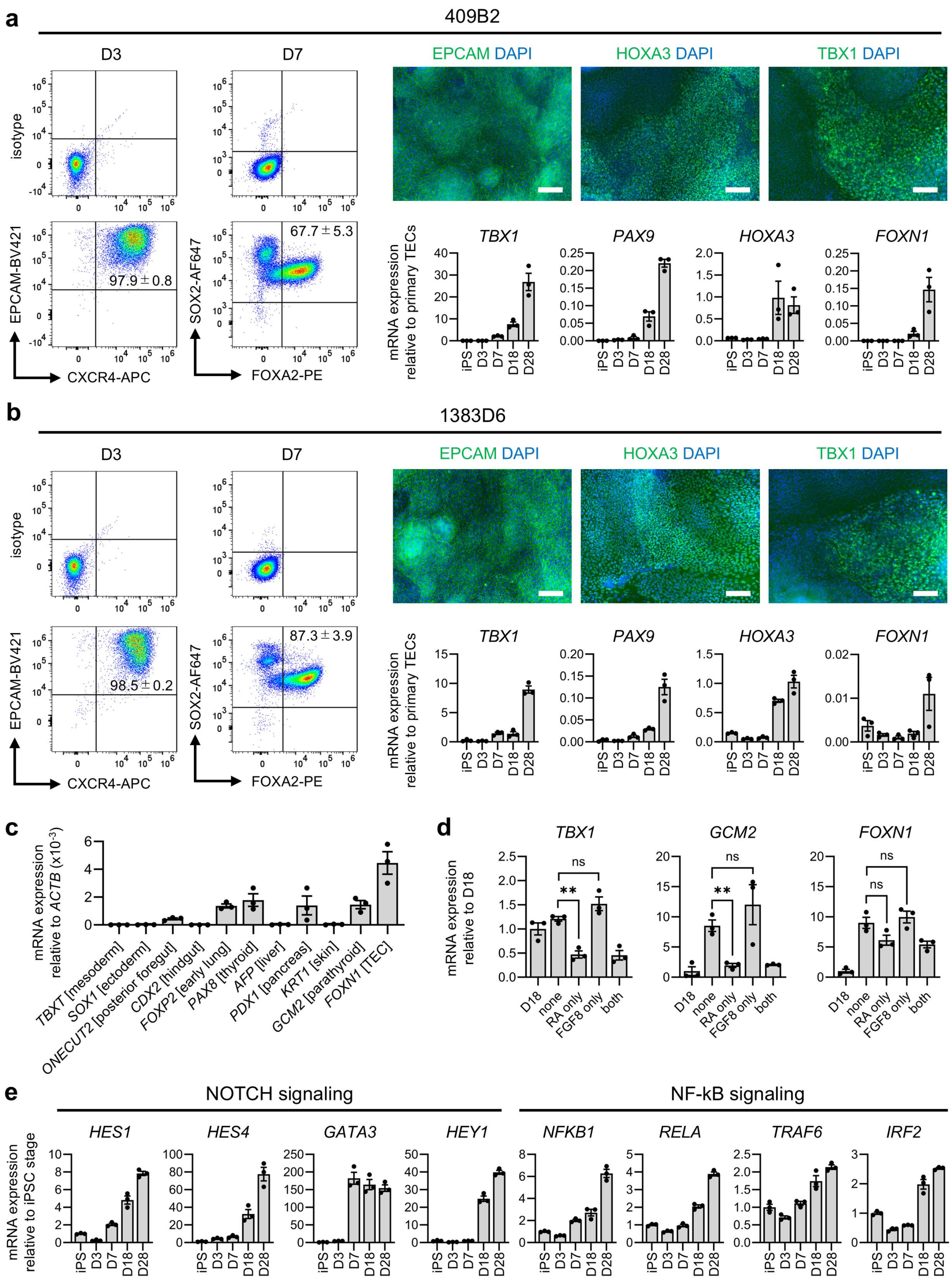

a
409B2
D3
D7
isotype
EPCAM-BV421
CXCR4-APC
SOX2-AF647
FOXA2-PE
97.9 ± 0.8
67.7 ± 5.3
EPCAM DAPI
HOXA3 DAPI
TBX1 DAPI
mRNA expression relative to primary TECs
TBX1
PAX9
HOXA3
FOXN1
iPS
D3
D7
D18
D28
b
1383D6
98.5 ± 0.2
87.3 ± 3.9
c
mRNA expression relative to ACTB (x10⁻³)
TBXT [mesoderm]
SOX1 [ectoderm]
ONECUT2 [posterior foregut]
CDX2 [hindgut]
FOXP2 [early lung]
PAX8 [thyroid]
AFP [liver]
PDX1 [pancreas]
KRT1 [skin]
GCM2 [parathyroid]
FOXN1 [TEC]
d
mRNA expression relative to D18
TBX1
GCM2
FOXN1
ns
**
none
RA only
FGF8 only
both
e
NOTCH signaling
NF-kB signaling
mRNA expression relative to iPSC stage
HES1
HES4
GATA3
HEY1
NFKB1
RELA
TRAF6
IRF2

Supplementary Figure 1. Validation of TEC induction with multiple iPSC lines and conditions

(a, b) Validation of thymic epithelial cell (TEC) induction in the 409B2 (a) and 1383D6 (b) induced pluripotent stem cell (iPSC) lines. Left, representative flow cytometry results with the respective isotype controls on D3 and D7; top right, representative images of immunostaining on D18; bottom right, mRNA expression of pharyngeal endoderm (PE) and third pharyngeal pouch (3rd PP) markers over time. Scale bars, 100 μm. D, day.

(c) mRNA expression of TEC and non-TEC lineage markers in the 201B7 iPSC line on D28. When the expression was not detected even after 45 cycles of amplification, the C_t value was set to a conservative overestimate of $C_t = 45$.

(d) mRNA expression of PE and 3rd PP markers in the 201B7 iPSC line on D28 after addition of 150 nM retinoic acid (RA), 50 ng/ml FGF8, or both from D18 to D28. The D18 control is also shown (leftmost bar).

(e) mRNA expression of NOTCH and NF-kB signaling molecules over time in the 201B7 iPSC line.

All results are from n=3 independent experiments and values indicate the mean ± SEM (standard error of the mean). Statistical significance was determined using unpaired two-sided t-tests (n.s. no significant difference, $*p < 0.05$, $**p < 0.01$).

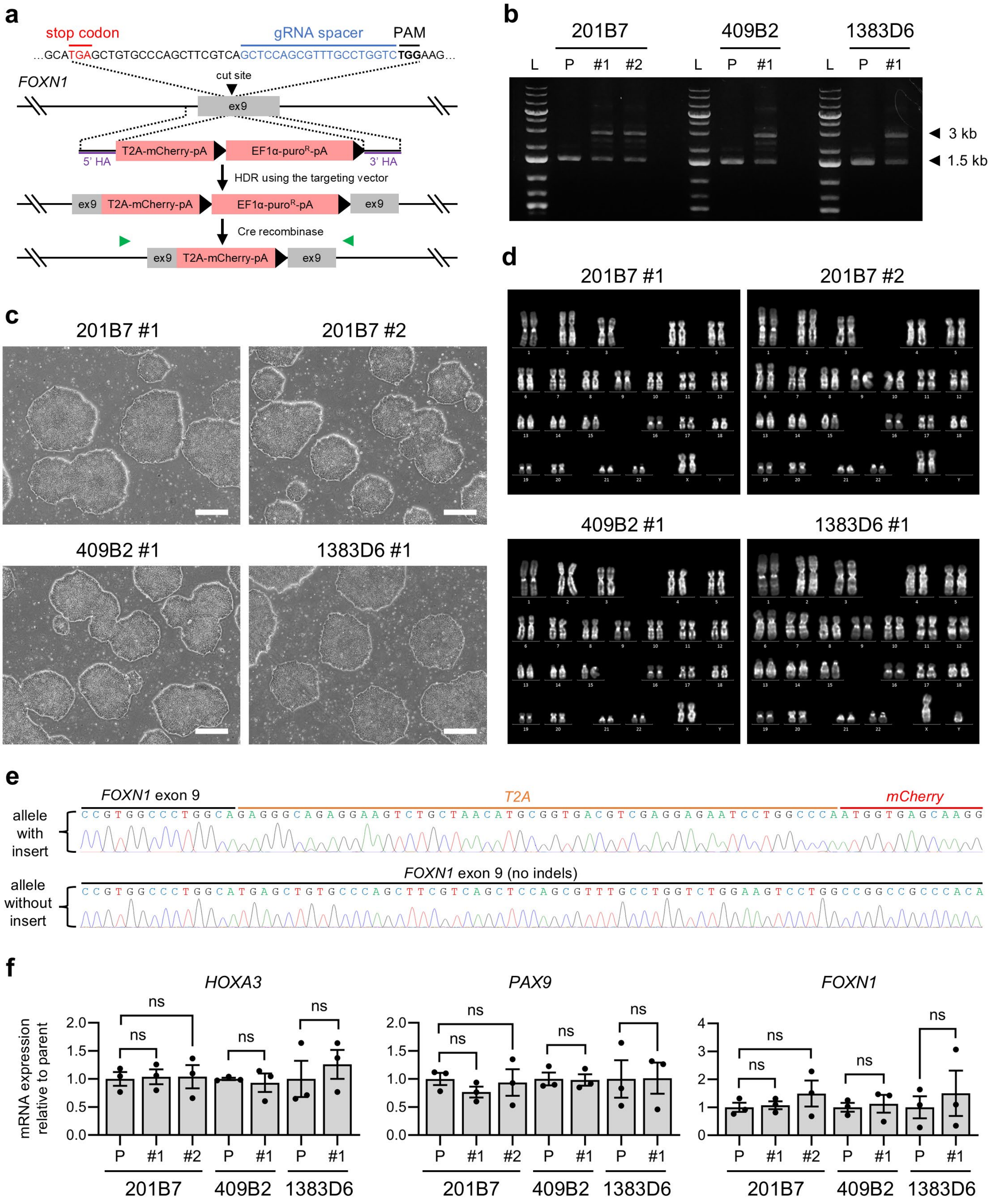
a
stop codon
gRNA spacer
PAM
...GCATGAGCTGTGCCCAGCTTCGTCAGCTCCAGCGTTTGCCTGGTCTGGAAG...
FOXN1
cut site
ex9
T2A-mCherry-pA
EF1α-puroR-pA
5' HA
3' HA
HDR using the targeting vector
Cre recombinase
b
201B7
409B2
1383D6
L P #1 #2
3 kb
1.5 kb
c
201B7 #1
201B7 #2
409B2 #1
1383D6 #1
d
e
FOXN1 exon 9
T2A
mCherry
allele with insert
allele without insert
FOXN1 exon 9 (no indels)
f
HOXA3
PAX9
FOXN1
mRNA expression relative to parent
ns
P #1 #2 P #1 P #1

Supplementary Figure 2. Establishment of *FOXN1^{mCherry}* reporters from multiple iPSC lines

(a) Schematic of the *FOXN1^{mCherry}* reporter design and targeting strategy. The guide RNA (gRNA) targets the 3' UTR near the stop codon located in exon 9 (ex9). The targeting vector contains the T2A-mCherry-pA sequences with a puromycin resistance (puro^{R}) cassette flanked by two loxP sites (black triangles), as well as 500-600 bp homology arms (HA). The floxed puromycin resistance cassette is removed using Cre recombinase. Successful clones are evaluated using the primers represented by green arrows, located just outside of the homology arms.

(b) Electrophoresis of PCR products amplified by the primer pair shown in (a). L, ladder; P, parent; #1 or #2, number of the reporter clone. 201B7 #1 is the main reporter clone shown in all main and supplementary figures except Supplementary Fig. 4. The presence of both the original 1.5 kb band and the new 3 kb band in the *FOXN1^{mCherry}* reporters indicates heterozygous insertion of the construct.

(c) Phase-contrast images of each *FOXN1^{mCherry}* reporter iPSC line. Scale bars, 500 μm.

(d) Karyotypes of the *FOXN1^{mCherry}* reporter iPSC lines, showing the same normal karyotype of their respective parent iPSC line. 201B7 and 409B2 reporters are 46,XX[8] and the 1383D6 reporter is 46,XY[8].

(e) Representative sequencing results of both alleles in the *FOXN1^{mCherry}* reporters around the stop codon, showing one allele with T2A-mCherry, and the other allele without insert or indels.

(f) mRNA expression of pharyngeal endoderm (PE) and third pharyngeal pouch (3rd PP) markers in each *FOXN1^{mCherry}* reporter relative to its respective parent line on day 28 of thymic epithelial cell (TEC) induction. Values indicate the mean ± SEM from n=3 independent experiments. Statistical significance was determined using unpaired two-sided t-tests (n.s. no significant difference, $*p < 0.05$).

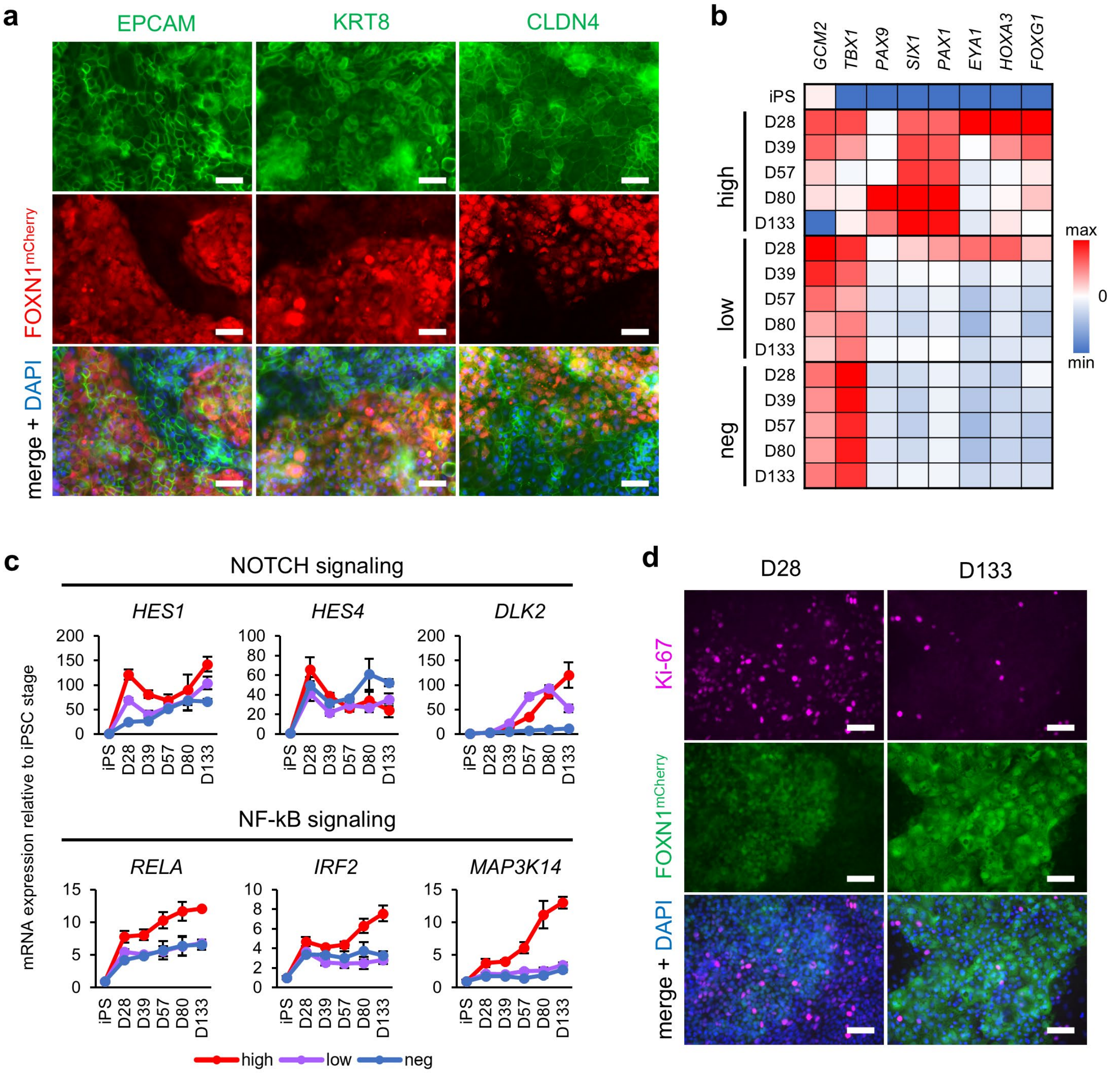
a
EPCAM
KRT8
CLDN4
FOXN1 mCherry
merge + DAPI
b
GCM2
TBX1
PAX9
SIX1
PAX1
EYA1
HOXA3
FOXG1
iPS
high
low
neg
D28
D39
D57
D80
D133
max
0
min
c
NOTCH signaling
HES1
HES4
DLK2
NF-kB signaling
RELA
IRF2
MAP3K14
mRNA expression relative to iPSC stage
high
low
neg
d
D28
D133
Ki-67
FOXN1 mCherry
merge + DAPI

Supplementary Figure 3. Self-directed signaling and TEC marker expression in induced cTEC- and mTEC-like cells

(a) Representative images of immunostaining on D80 from n=6 independent experiments. Scale bars, 50 μm.

(b) Heatmap of the mean $\log_2$ fold change in mRNA expression relative to primary TECs in the mCherry^{high}, mCherry^{low}, and mCherry^{-} (neg) populations over time from n=6 (D0 to D80) or n=3 (D133) independent experiments.

(c) mRNA expression of NOTCH and NF-kB signaling molecules in the mCherry^{high}, mCherry^{low}, and mCherry^{-} populations over time. All values indicate the mean ± SEM from n=3 independent experiments.

(d) Representative images of immunostaining on D28 and D133 from n=4 independent experiments. Scale bars, 50 μm.

All results were obtained using the 201B7 *FOXN1^{mCherry}* reporter iPSC line. D, day; TEC, thymic epithelial cell; cTEC, cortical TEC; mTEC, medullary TEC.

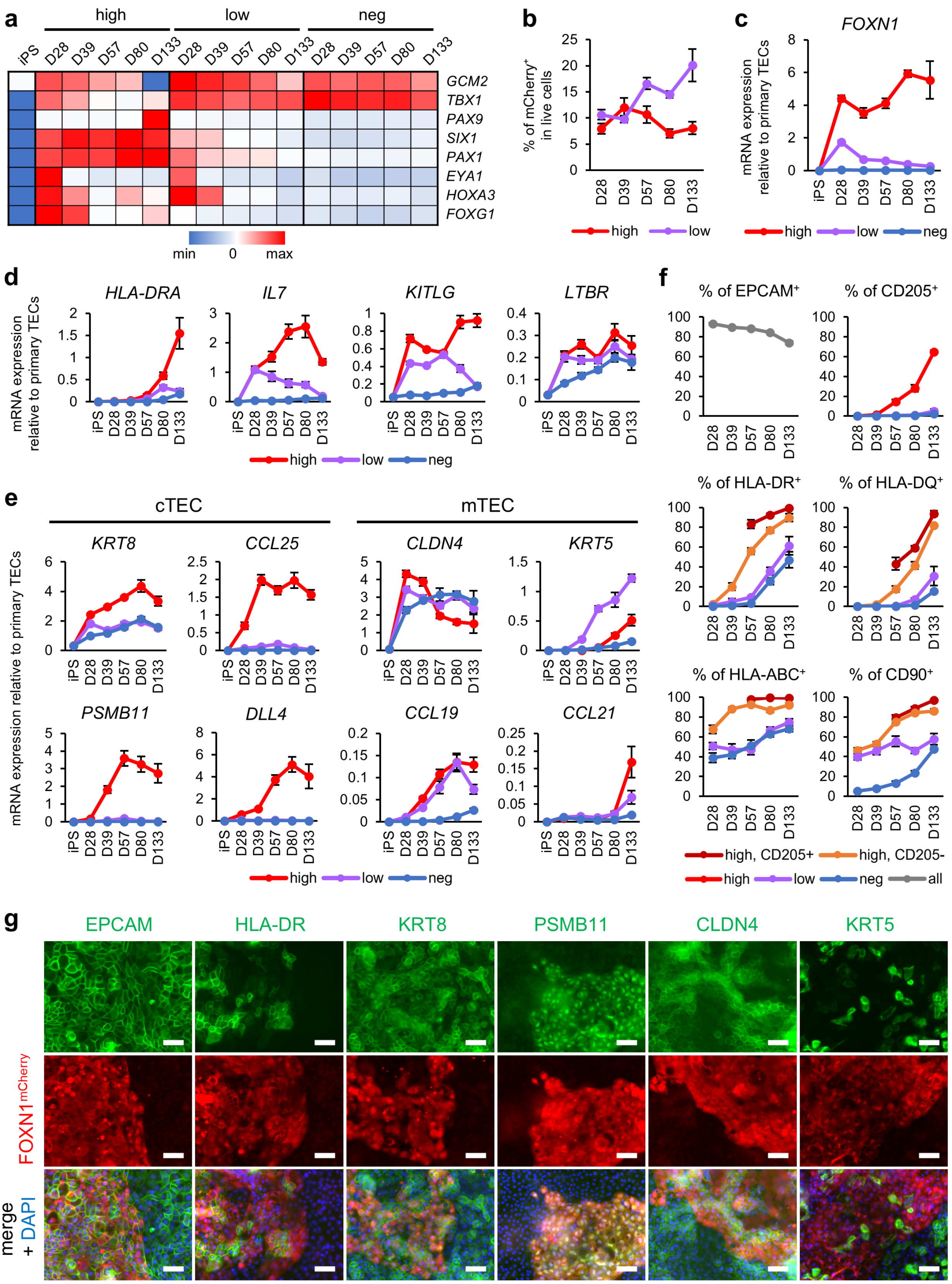
a
high
low
neg
iPS
D28
D39
D57
D80
D133
GCM2
TBX1
PAX9
SIX1
PAX1
EYA1
HOXA3
FOXG1
min
0
max
b
% of mCherry+ in live cells
high
low
c
FOXN1
mRNA expression relative to primary TECs
high
low
neg
d
HLA-DRA
IL7
KITLG
LTBR
mRNA expression relative to primary TECs
e
cTEC
mTEC
KRT8
CCL25
CLDN4
KRT5
PSMB11
DLL4
CCL19
CCL21
mRNA expression relative to primary TECs
f
% of EPCAM+
% of CD205+
% of HLA-DR+
% of HLA-DQ+
% of HLA-ABC+
% of CD90+
high, CD205+
high, CD205-
all
g
EPCAM
HLA-DR
KRT8
PSMB11
CLDN4
KRT5
FOXN1 mCherry
merge + DAPI

Supplementary Figure 4. Expression of TEC lineage and functional markers in a second 201B7 *FOXN1^{mCherry}* reporter line

(a) Heatmap of the mean log_2 fold change in mRNA expression relative to primary TECs in the mCherry^{high}, mCherry^{low}, and mCherry^{-} (neg) populations over time from n=6 (D0 to D80) or n=3 (D133) independent experiments.

(b) Quantification of the percentage of mCherry^{high} and mCherry^{low} cells using flow cytometry.

(c-e) mRNA expression of TEC markers in the mCherry^{high}, mCherry^{low}, and mCherry^{-} populations over time.

(f) Quantification of flow cytometry results over time, with EPCAM^{+} cells (top left), CD205^{+} cells in each of the mCherry^{high}, mCherry^{low}, and mCherry^{-} populations (top right), and HLA-DR^{+}, HLA-DQ^{+}, HLA-ABC^{+}, and CD90^{+} cells in the mCherry^{high}CD205^{+}, mCherry^{high}CD205^{-}, mCherry^{low}, and mCherry^{-} populations (middle and bottom). Except for the top left panel, all populations are gated on EPCAM^{+}.

(g) Representative images of immunostaining on D80 from n=6 independent experiments. Scale bars, 50 μm.

All results were obtained using the 201B7 *FOXN1^{mCherry}* reporter #2 iPSC line. In (b) to (f), all values indicate the mean ± SEM from n=6 (D0 to D80) or n=3 (D133) independent experiments. D, day; TEC, thymic epithelial cell; cTEC, cortical TEC; mTEC, medullary TEC.

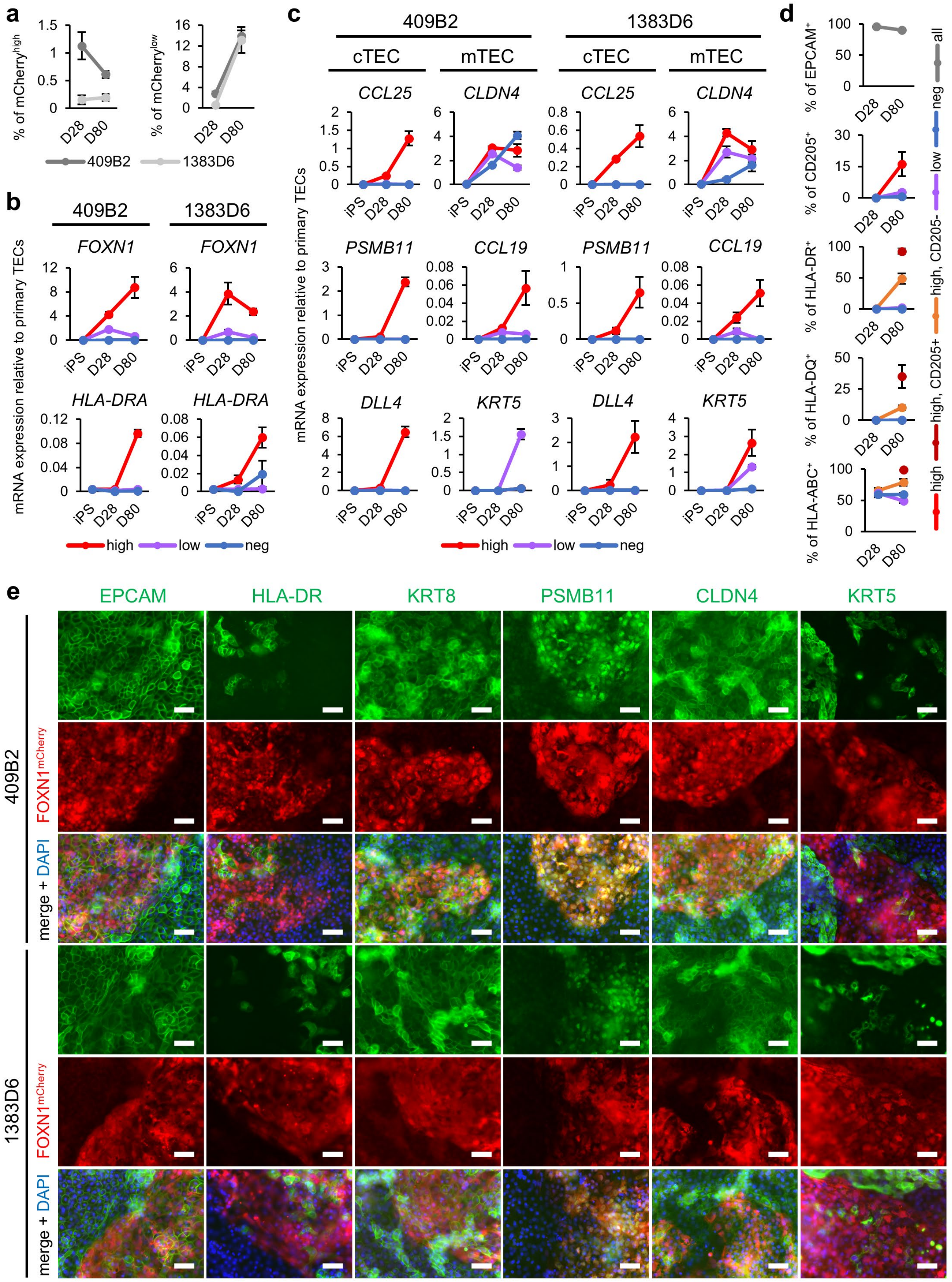

a
% of mCherry^high
% of mCherry^low
D28
D80
409B2
1383D6
b
409B2
1383D6
FOXN1
HLA-DRA
mRNA expression relative to primary TECs
iPS
high
low
neg
c
409B2
1383D6
cTEC
mTEC
CCL25
CLDN4
PSMB11
CCL19
DLL4
KRT5
d
% of EPCAM+
% of CD205+
% of HLA-DR+
% of HLA-DQ+
% of HLA-ABC+
all
neg
low
high, CD205-
high, CD205+
high
e
EPCAM
HLA-DR
KRT8
PSMB11
CLDN4
KRT5
FOXN1mCherry
merge + DAPI

Supplementary Figure 5. Expression of TEC lineage and functional markers in 409B2 and 1383D6 *FOXN1*^{mCherry} reporter lines

(a) Quantification of the percentage of mCherry^{high} and mCherry^{low} cells in live cells at each time point using flow cytometry. All values indicate the mean ± SEM from n=7 (409B2) or n=4 (1383D6) independent experiments.

(b, c) mRNA expression of TEC markers in the mCherry^{high}, mCherry^{low}, and mCherry^{-} (neg) populations over time. All values indicate the mean ± SEM from n=3 independent experiments.

(d) Quantification of flow cytometry results on D28 and D80, with EPCAM^{+} cells (top), CD205^{+} cells in each of the mCherry^{high}, mCherry^{low}, and mCherry^{-} populations (second from top), and HLA-DR^{+}, HLA-DQ^{+}, and HLA-ABC^{+} cells in the mCherry^{high}CD205^{+}, mCherry^{high}CD205^{-}, mCherry^{low}, and mCherry^{-} populations (bottom three). Except for the top panel, all populations are gated on EPCAM^{+}. All values indicate the mean ± SEM from n=7 independent experiments using the 409B2 line.

(e) Representative images of immunostaining on D80 from n=3 independent experiments. Scale bars, 50 µm.

All results were obtained using the 409B2 and 1383D6 *FOXN1*^{mCherry} reporter lines as indicated within each panel. D, day; TEC, thymic epithelial cell; cTEC, cortical TEC; mTEC, medullary TEC.

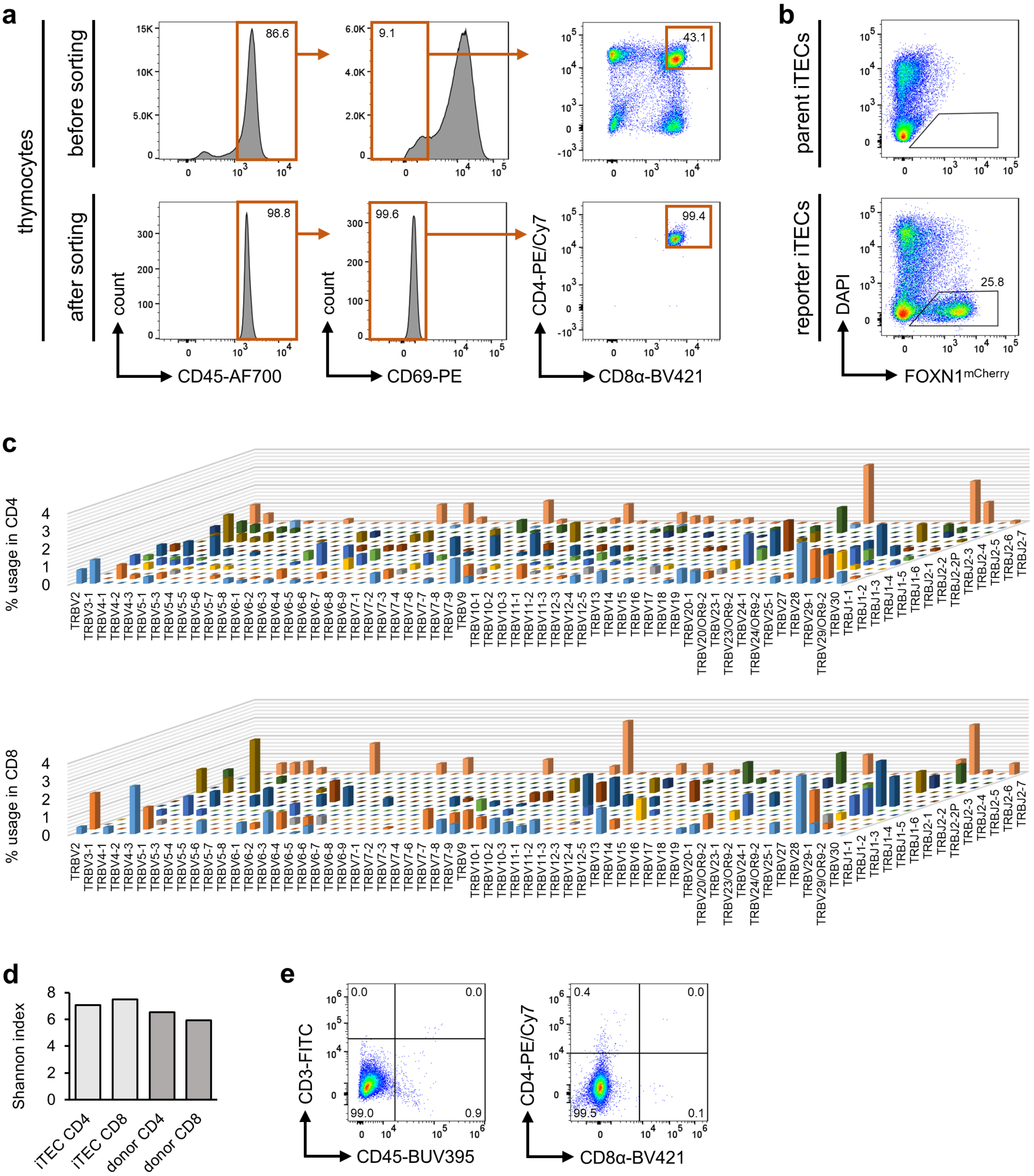
a
thymocytes
before sorting
after sorting
86.6
9.1
43.1
98.8
99.6
99.4
count
CD45-AF700
CD69-PE
CD4-PE/Cy7
CD8α-BV421
b
parent iTECs
reporter iTECs
25.8
DAPI
FOXN1mCherry
c
% usage in CD4
% usage in CD8
d
Shannon index
iTEC CD4
iTEC CD8
donor CD4
donor CD8
e
0.0
0.0
99.0
0.9
0.4
0.0
99.5
0.1
CD3-FITC
CD45-BUV395
CD4-PE/Cy7
CD8α-BV421

Supplementary Figure 6. TCR diversity in donor and iTEC-supported CD4$^+$ and CD8$^+$ T cells

(a) Gating for the isolation of double-positive (DP) thymocytes for organoid co-culture (top) and re-analysis of the isolated DP thymocytes as a day 0 control (bottom), representative of n=4 independent experiments.

(b) Gating for the isolation of day 37 mCherry$^+$ induced thymic epithelial cells (iTEC) for organoid co-culture, representative of n=4 independent experiments.

(c) Gene usage of *TRBV* and *TRBJ* in CD4$^+$ (top) and CD8$^+$ (bottom) single-positive (SP) thymocytes from n=1 pediatric donor. These SP thymocytes are from the same donor as the DP thymocytes that were used for iTEC co-culture and subsequent TCR repertoire analysis in Fig. 4e.

(d) Shannon index calculated using TCR repertoires of CD4$^+$ and CD8$^+$ SP thymocytes derived from iTEC co-culture or pediatric donor from Fig. 4e and Supplementary Fig. 6c.

(e) Representative flow cytometry results after 14 days of mCherry$^-$ iTEC organoid co-culture with DP thymocytes from n=4 independent experiments.

All iTEC results were obtained using the 201B7 *FOXN1^{mCherry}* reporter iPSC line.

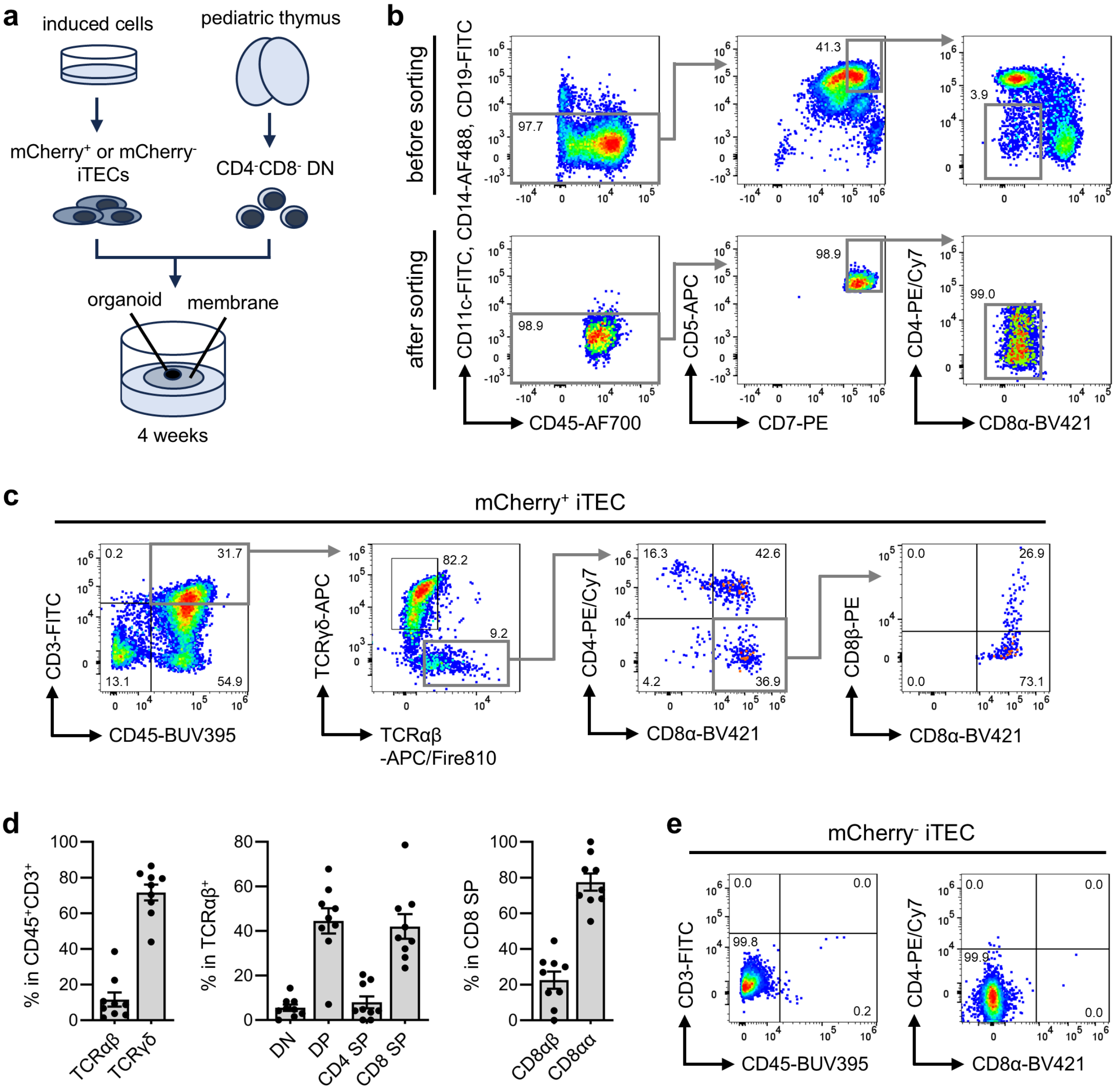
a
induced cells
pediatric thymus
mCherry+ or mCherry- iTECs
CD4-CD8- DN
organoid
membrane
4 weeks
b
before sorting
after sorting
CD11c-FITC, CD14-AF488, CD19-FITC
CD45-AF700
CD5-APC
CD7-PE
CD4-PE/Cy7
CD8α-BV421
97.7
41.3
3.9
98.9
98.9
99.0
c
mCherry+ iTEC
CD3-FITC
CD45-BUV395
0.2
31.7
13.1
54.9
TCRγδ-APC
TCRαβ -APC/Fire810
82.2
9.2
CD4-PE/Cy7
CD8α-BV421
16.3
42.6
4.2
36.9
CD8β-PE
CD8α-BV421
0.0
26.9
0.0
73.1
d
% in CD45+CD3+
TCRαβ
TCRγδ
% in TCRαβ+
DN
DP
CD4 SP
CD8 SP
% in CD8 SP
CD8αβ
CD8αα
e
mCherry- iTEC
CD3-FITC
CD45-BUV395
0.0
0.0
99.8
0.2
CD4-PE/Cy7
CD8α-BV421
0.0
0.0
99.9
0.0

Supplementary Figure 7. Generation of DP and SP thymocytes from the DN stage in iTEC co-culture

(a) Schematic of induced thymic epithelial cell (iTEC) co-culture with double-negative (DN) thymocytes.

(b) Gating for the isolation of DN thymocytes for organoid co-culture (top) and re-analysis of the isolated DN thymocytes as a day 0 control (bottom), representative of n=3 independent experiments.

(c, d) Representative flow cytometry results (c) and quantification of each population (d) after 4 weeks of mCherry$^+$ iTEC organoid co-culture with DN thymocytes, with values in (d) indicating the mean ± SEM from n=9 independent experiments.

(e) Representative flow cytometry results after 4 weeks of mCherry$^-$ iTEC organoid co-culture with DN thymocytes from n=4 independent experiments.

All iTEC results were obtained using the 201B7 *FOXN1*^{mCherry} reporter iPSC line. DP, double-positive; SP, single-positive.

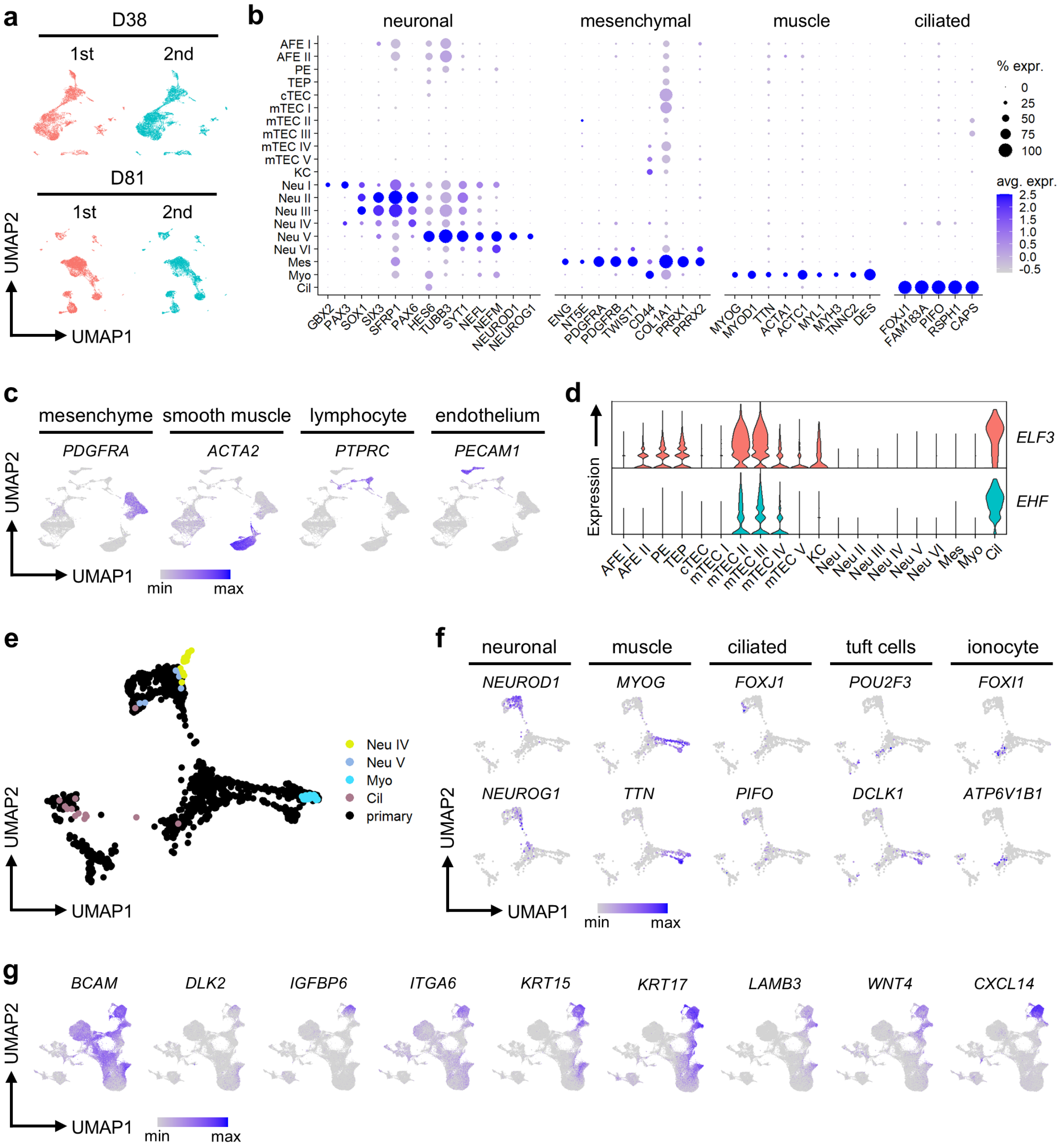

a
D38
1st
2nd
D81
1st
2nd
UMAP2
UMAP1
b
neuronal
mesenchymal
muscle
ciliated
AFE I
AFE II
PE
TEP
cTEC
mTEC I
mTEC II
mTEC III
mTEC IV
mTEC V
KC
Neu I
Neu II
Neu III
Neu IV
Neu V
Neu VI
Mes
Myo
Cil
GBX2 PAX3 SOX1 SIX3 SFRP1 PAX6 HES6 TUBB3 SYT1 NEFL NEFM NEUROD1 NEUROG1
ENG NT5E PDGFRA PDGFRB TWIST1 CD44 COL1A1 PRRX1 PRRX2
MYOG MYOD1 TTN ACTA1 ACTC1 MYL1 MYH3 TNNC2 DES
FOXJ1 FAM183A PIFO RSPH1 CAPS
% expr.
0
25
50
75
100
avg. expr.
2.5
2.0
1.5
1.0
0.5
0.0
-0.5
c
mesenchyme
smooth muscle
lymphocyte
endothelium
PDGFRA
ACTA2
PTPRC
PECAM1
UMAP2
UMAP1
min
max
d
Expression
ELF3
EHF
AFE I AFE II PE TEP cTEC mTEC I mTEC II mTEC III mTEC IV mTEC V KC Neu I Neu II Neu III Neu IV Neu V Neu VI Mes Myo Cil
e
Neu IV
Neu V
Myo
Cil
primary
UMAP2
UMAP1
f
neuronal
muscle
ciliated
tuft cells
ionocyte
NEUROD1
MYOG
FOXJ1
POU2F3
FOXI1
NEUROG1
TTN
PIFO
DCLK1
ATP6V1B1
UMAP2
UMAP1
min
max
g
BCAM
DLK2
IGFBP6
ITGA6
KRT15
KRT17
LAMB3
WNT4
CXCL14
UMAP2
UMAP1
min
max

Supplementary Figure 8. Expression of diverse lineage markers in induced cells and primary thymic stroma

(a) UMAP of n=2 independent experiments on D38 (top) and D81 (bottom) after merging the respective datasets, showing highly similar population distributions. “1st” is the main induction from Fig. 5 to 7, while “2nd” is the additional induction. D, day.

(b) Dot plot of non-TEC lineage marker gene expression in induced cells. AFE, anterior foregut endoderm; PE, pharyngeal endoderm; TEP, thymic epithelial progenitor; TEC, thymic epithelial cell; cTEC, cortical TEC; mTEC, medullary TEC; KC, keratinized cell; Neu, neuron; Myo, myocyte; Mes, mesenchyme; Cil, ciliated; % expr., percentage of cells in which the gene is expressed; avg. expr., average expression.

(c) Expression of markers of mesenchyme, smooth muscle, lymphocytes, and endothelium in primary thymic stroma on UMAP.

(d) Violin plot of *ELF3* and *EHF* expression in each induced cluster.

(e) UMAP of induced D133 non-TEC clusters on primary mTEC-CLDN3/4 and mTEC-mim clusters (black) from Fig. 6a.

(f) Expression of markers of various lineages in primary mTEC-CLDN3/4 and mTEC-mim clusters on UMAP, indicating different types of mimetic mTECs.

(g) Expression of mcTEC (intertypical TEC) and mTEC marker genes in induced cells on UMAP.

All results were obtained using primary thymic stroma from n=3 pediatric donors and the 201B7 $\textit{FOXN1}^{\textit{mCherry}}$ reporter iPSC line.

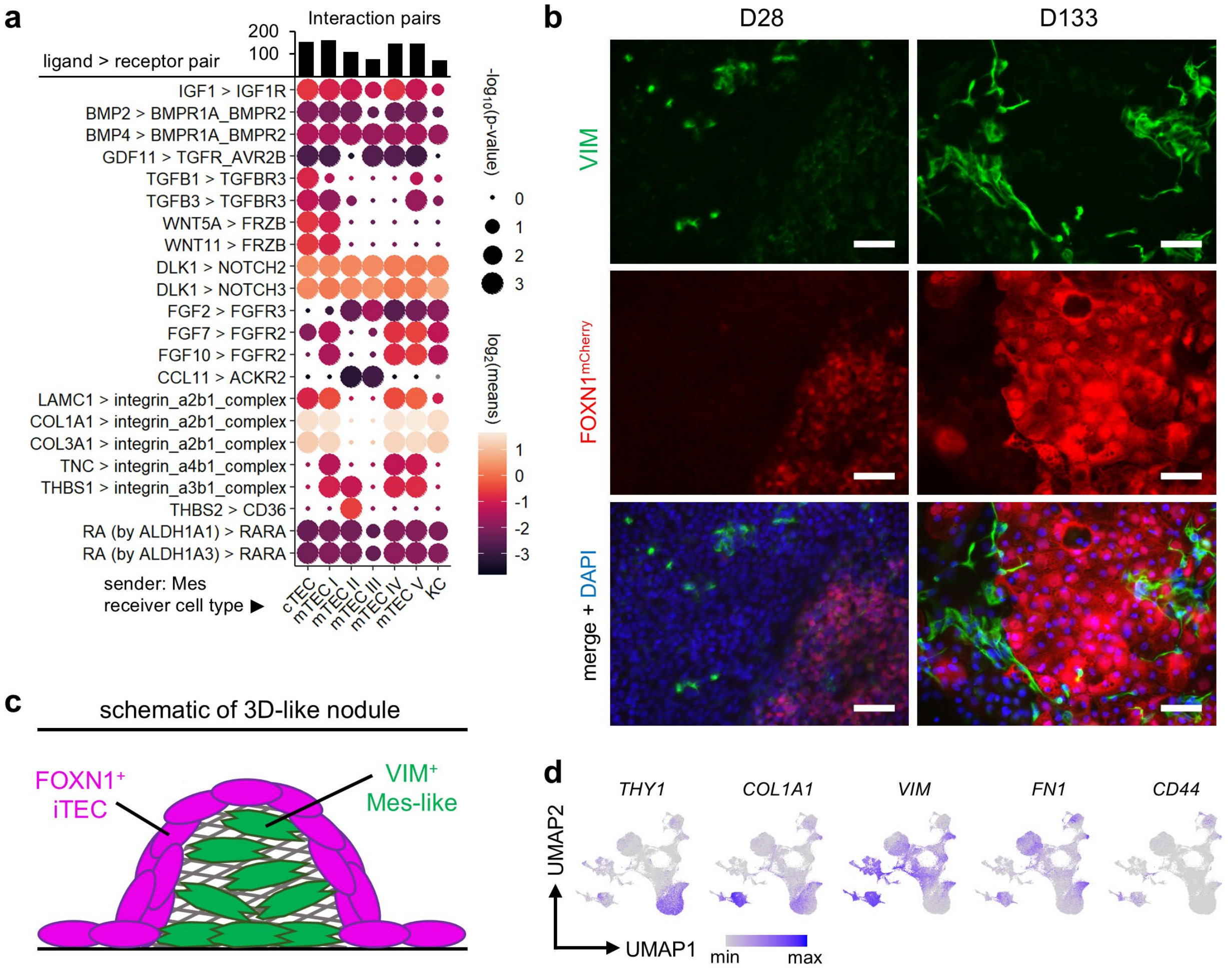

a
Interaction pairs
200
100
ligand > receptor pair
IGF1 > IGF1R
BMP2 > BMPR1A_BMPR2
BMP4 > BMPR1A_BMPR2
GDF11 > TGFR_AVR2B
TGFB1 > TGFBR3
TGFB3 > TGFBR3
WNT5A > FRZB
WNT11 > FRZB
DLK1 > NOTCH2
DLK1 > NOTCH3
FGF2 > FGFR3
FGF7 > FGFR2
FGF10 > FGFR2
CCL11 > ACKR2
LAMC1 > integrin_a2b1_complex
COL1A1 > integrin_a2b1_complex
COL3A1 > integrin_a2b1_complex
TNC > integrin_a4b1_complex
THBS1 > integrin_a3b1_complex
THBS2 > CD36
RA (by ALDH1A1) > RARA
RA (by ALDH1A3) > RARA
sender: Mes
receiver cell type ▶
cTEC
mTEC I
mTEC II
mTEC III
mTEC IV
mTEC V
KC
-log10(p-value)
0
1
2
3
log2(means)
1
0
-1
-2
-3
b
D28
D133
VIM
FOXN1mCherry
merge + DAPI
c
schematic of 3D-like nodule
FOXN1+ iTEC
VIM+ Mes-like
d
THY1
COL1A1
VIM
FN1
CD44
UMAP2
UMAP1
min
max

Supplementary Figure 9. Localization and potential interactions of mesenchyme-like cells in iTEC culture

(a) Dot plot of potential ligand-receptor interactions between the induced Mes cluster (sender) and AFE-derived terminal clusters (receivers) on D133 as predicted by CellPhoneDB. The number of significant interaction pairs ($p < 0.05$) is shown in black bars above each population. D, day; AFE, anterior foregut endoderm; TEC, thymic epithelial cell; cTEC, cortical TEC; mTEC, medullary TEC; KC, keratinized cell; Mes, mesenchyme.

(b) Representative images of immunostaining on D28 and D133 from n=3 independent experiments. Scale bars, 50 µm.

(c) Schematic of 3D-like nodule on D133 of TEC induction visualized in Supplementary Video 3, showing the VIM^{+} core below the FOXN1^{+} layer. iTEC, induced TEC.

(d) Expression of mesenchymal marker genes in induced cells on UMAP.

All results were obtained using the 201B7 *FOXN1*^{mCherry} reporter iPSC line.

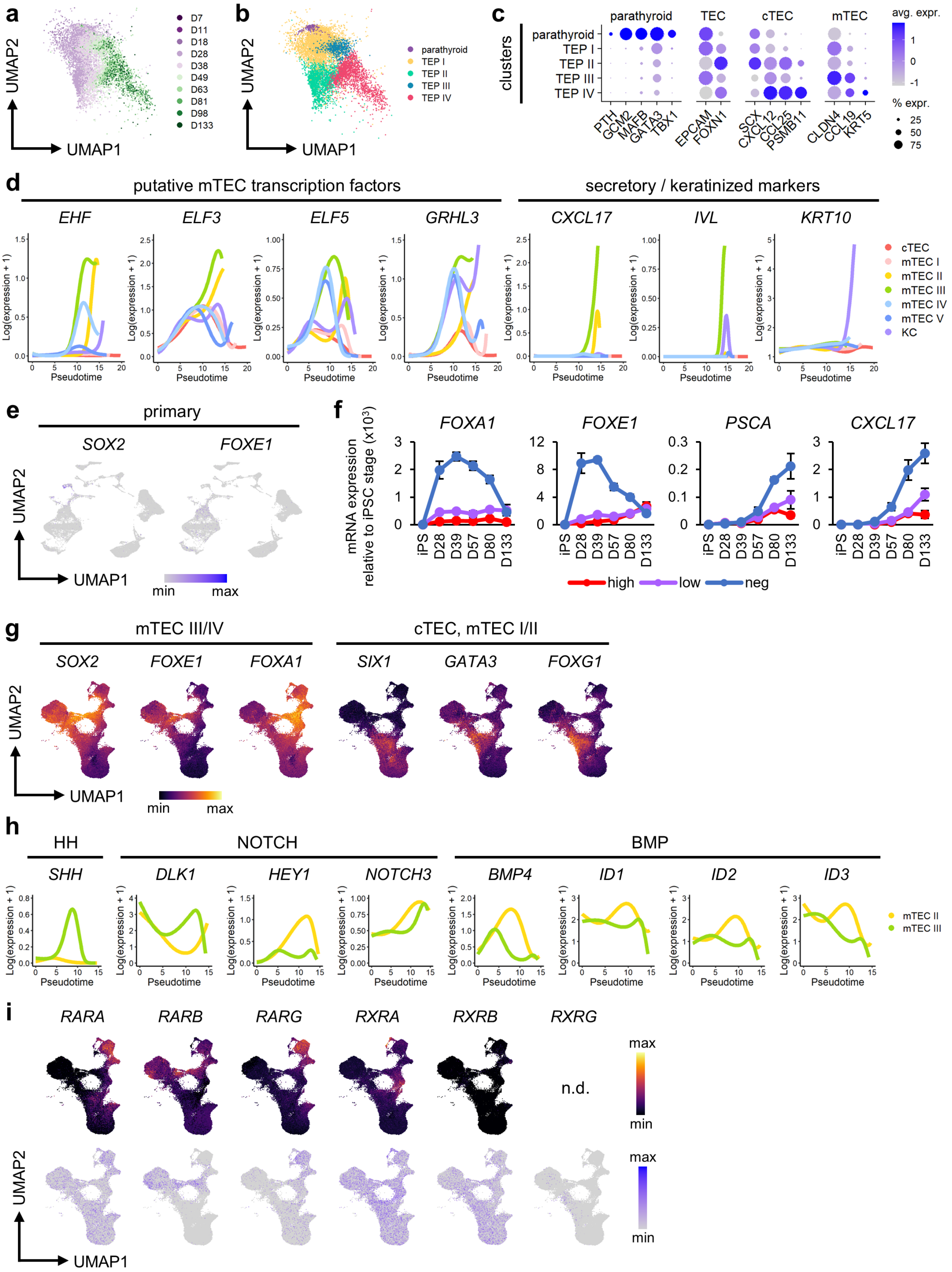

a
UMAP2
UMAP1
D7
D11
D18
D28
D38
D49
D63
D81
D98
D133
b
parathyroid
TEP I
TEP II
TEP III
TEP IV
c
clusters
parathyroid
TEC
cTEC
mTEC
avg. expr.
% expr.
PTH
GCM2
MAFB
GATA3
TBX1
EPCAM
FOXN1
SCX
CXCL12
CCL25
PSMB11
CLDN4
CCL19
KRT5
d
putative mTEC transcription factors
secretory / keratinized markers
EHF
ELF3
ELF5
GRHL3
CXCL17
IVL
KRT10
Log(expression + 1)
Pseudotime
cTEC
mTEC I
mTEC II
mTEC III
mTEC IV
mTEC V
KC
e
primary
SOX2
FOXE1
min
max
f
FOXA1
FOXE1
PSCA
CXCL17
mRNA expression relative to iPSC stage (x10³)
iPS
D28
D39
D57
D80
D133
high
low
neg
g
mTEC III/IV
cTEC, mTEC I/II
SOX2
FOXE1
FOXA1
SIX1
GATA3
FOXG1
h
HH
NOTCH
BMP
SHH
DLK1
HEY1
NOTCH3
BMP4
ID1
ID2
ID3
mTEC II
mTEC III
i
RARA
RARB
RARG
RXRA
RXRB
RXRG
n.d.

Supplementary Figure 10. Characterization of induced TEPs and early lineage divergence

(a) UMAP of the isolated TEP cluster from Fig. 7a colored by induction time point.

(b) Subclusters of induced TEPs on UMAP.

(c) Dot plot of parathyroid and TEC lineage marker gene expression in induced TEP subclusters. % expr., percentage of cells in which the gene is expressed; avg. expr., average expression.

(d) Smoothed gene expression of putative mTEC transcription factors and secretory or keratinized cell markers along pseudotime in each induced lineage.

(e) Expression of *SOX2* and *FOXE1* in primary thymic stroma on UMAP.

(f) mRNA expression of induced mTEC III and secretory cell markers in the sorted $mCherry^{high}$, $mCherry^{low}$, and $mCherry^{-}$ (neg) populations over time by qPCR. All values indicate the mean ± SEM from n=3 independent experiments.

(g) Scaled AUC (Area Under the Curve) scores from SCENIC in the induced AFE-derived lineages on UMAP, showing regulons with high activity in the induced mTEC III/IV (left) and cTEC, mTEC I/II (right) lineages.

(h) Smoothed gene expression of differentially expressed genes of major developmental pathways along pseudotime in the induced mTEC II and mTEC III lineages.

(i) Scaled AUC scores from SCENIC (top) and gene expression (bottom) of retinoic acid (RA) receptors in the induced AFE-derived lineages on UMAP. n.d., not detected.

All results were obtained using primary thymic stroma from n=3 pediatric donors and the 201B7 $FOXN1^{mCherry}$ reporter iPSC line. D, day; AFE, anterior foregut endoderm; TEP, thymic epithelial progenitor; TEC, thymic epithelial cell; cTEC, cortical TEC; mTEC, medullary TEC; KC, keratinized cell.

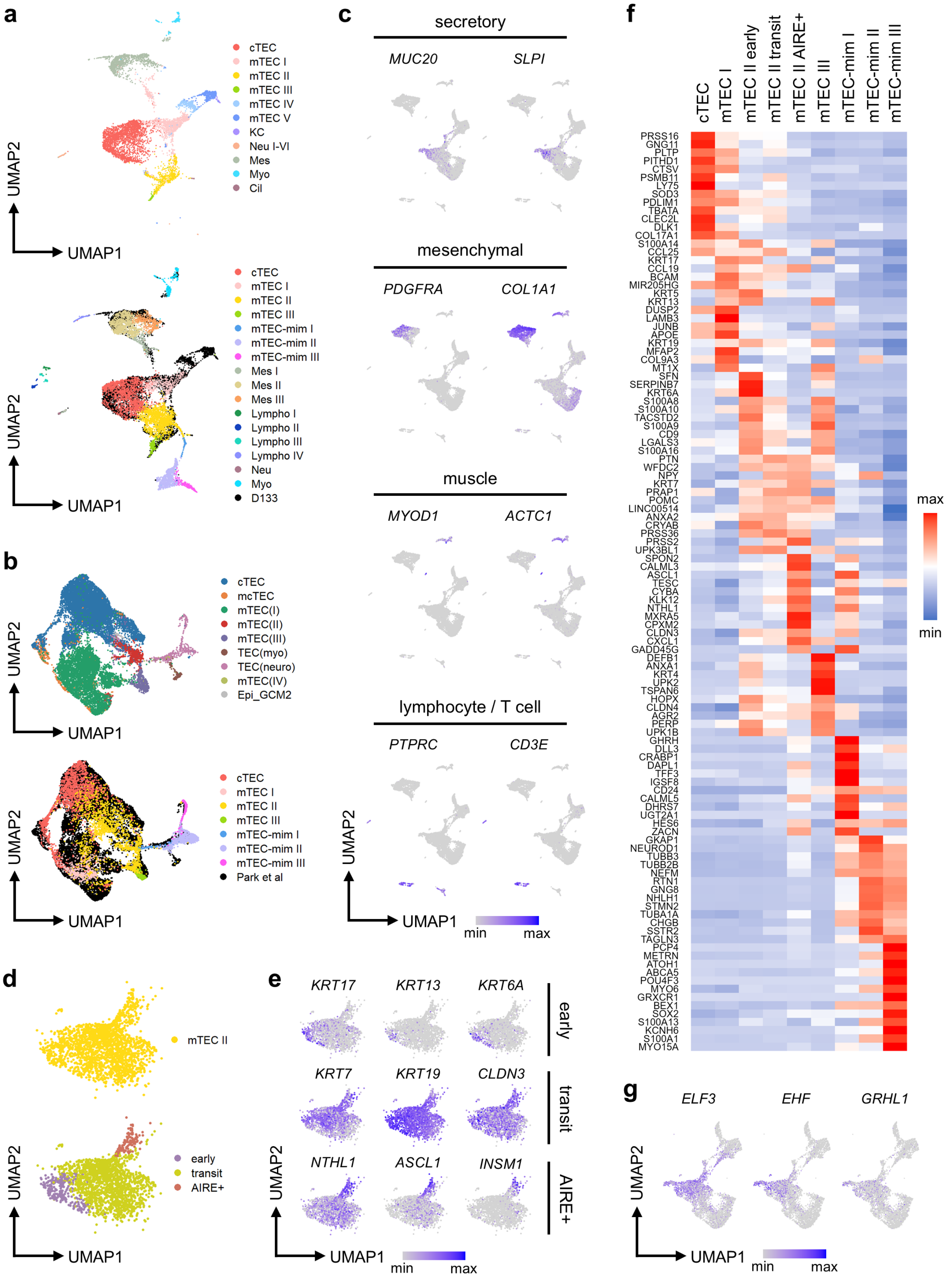

a
cTEC
mTEC I
mTEC II
mTEC III
mTEC IV
mTEC V
KC
Neu I-VI
Mes
Myo
Cil
UMAP2
UMAP1
cTEC
mTEC I
mTEC II
mTEC III
mTEC-mim I
mTEC-mim II
mTEC-mim III
Mes I
Mes II
Mes III
Lympho I
Lympho II
Lympho III
Lympho IV
Neu
Myo
D133
UMAP2
UMAP1
b
cTEC
mcTEC
mTEC(I)
mTEC(II)
mTEC(III)
TEC(myo)
TEC(neuro)
mTEC(IV)
Epi_GCM2
UMAP2
UMAP1
cTEC
mTEC I
mTEC II
mTEC III
mTEC-mim I
mTEC-mim II
mTEC-mim III
Park et al
UMAP2
UMAP1
c
secretory
MUC20
SLPI
mesenchymal
PDGFRA
COL1A1
muscle
MYOD1
ACTC1
lymphocyte / T cell
PTPRC
CD3E
UMAP2
UMAP1
min
max
d
mTEC II
early
transit
AIRE+
UMAP2
UMAP1
e
KRT17
KRT13
KRT6A
early
KRT7
KRT19
CLDN3
transit
NTHL1
ASCL1
INSM1
AIRE+
UMAP2
UMAP1
min
max
f
cTEC
mTEC I
mTEC II early
mTEC II transit
mTEC II AIRE+
mTEC III
mTEC-mim I
mTEC-mim II
mTEC-mim III
PRSS16
GNG11
PLTP
PITHD1
CTSV
PSMB11
LY75
SOD3
PDLIM1
TBATA
CLEC2L
DLK1
COL17A1
S100A14
CCL25
KRT17
CCL19
BCAM
MIR205HG
KRT5
KRT13
DUSP2
LAMB3
JUNB
APOE
KRT19
MFAP2
COL9A3
MT1X
SFN
SERPINB7
KRT6A
S100A8
S100A10
TACSTD2
S100A9
CD9
LGALS3
S100A16
PTN
WFDC2
NPY
KRT7
PRAP1
POMC
LINC00514
ANXA2
CRYAB
PRSS36
PRSS2
UPK3BL1
SPON2
CALML3
ASCL1
TESC
CYBA
KLK12
NTHL1
MXRA5
CPXM2
CLDN3
CXCL1
GADD45G
DEFB1
ANXA1
KRT4
UPK2
TSPAN6
HOPX
CLDN4
AGR2
PERP
UPK1B
GHRH
DLL3
CRABP1
DAPL1
TFF3
IGSF8
CD24
CALML5
DHRS7
UGT2A1
HES6
ZACN
GKAP1
NEUROD1
TUBB3
TUBB2B
NEFM
RTN1
GNG8
NHLH1
STMN2
TUBA1A
CHGB
SSTR2
TAGLN3
PCP4
METRN
ATOH1
ABCA5
POU4F3
MYO6
GRXCR1
BEX1
SOX2
S100A13
KCNH6
S100A1
MYO15A
max
min
g
ELF3
EHF
GRHL1
UMAP2
UMAP1
min
max

Supplementary Figure 11. Characterization of iTECs after thymocyte co-culture

(a) UMAP of the iTEC/thymocyte co-culture cells from Fig. 8c integrated with the D133 induced cells from Fig. 5b, showing the clustering of the D133 induced cells alone (top) and the overlay of the iTEC/thymocyte co-culture cells (bottom).

(b) UMAP of the co-cultured iTECs from Fig. 8c integrated with the Park et al. dataset of human thymic epithelium, showing the clustering of the Park et al. dataset alone (top) and the overlay of the co-cultured iTECs (bottom).

(c) Expression of marker genes of various cell types in the iTEC/thymocyte co-culture cells from Fig. 8c on UMAP.

(d) UMAP of the isolated mTEC II cluster from Fig. 8c (top) and unsupervised subclustering (bottom).

(e) Expression of selected marker genes from each mTEC II subcluster on UMAP.

(f) Heatmap of the average expression of the top 15 differentially expressed marker genes in each cluster of co-cultured iTECs.

(g) Expression of markers of mTEC development in the *EPCAM*$^{+}$ clusters on UMAP.

All results were obtained using the 201B7 *FOXN1*^{mCherry} reporter iPSC line co-cultured with primary double-positive (DP) thymocytes. D, day; TEC, thymic epithelial cell; cTEC, cortical TEC; mTEC, medullary TEC; iTEC, induced TEC.

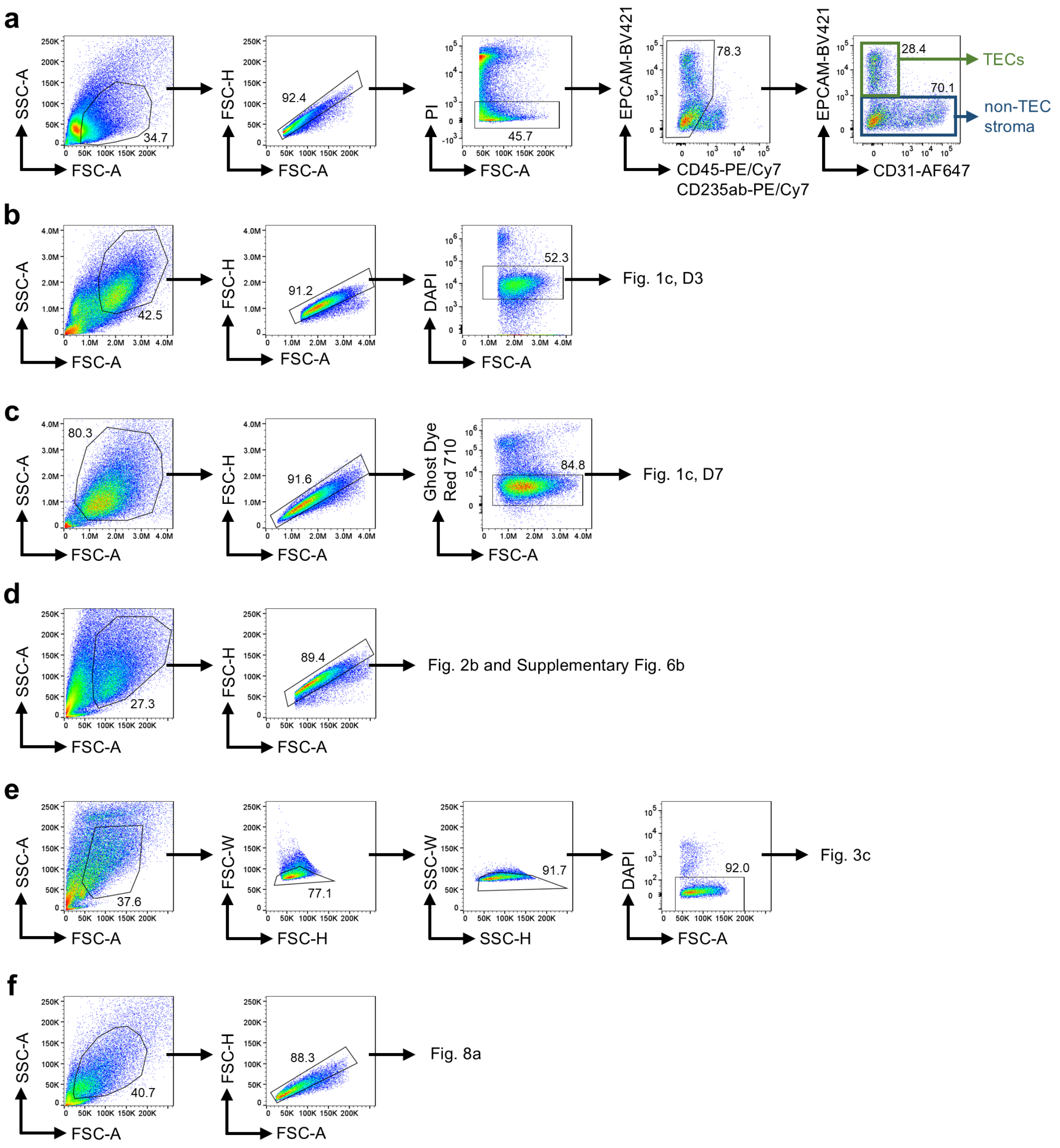
a
SSC-A
FSC-A
34.7
FSC-H
92.4
PI
45.7
EPCAM-BV421
CD45-PE/Cy7
CD235ab-PE/Cy7
78.3
CD31-AF647
28.4
70.1
TECs
non-TEC stroma
b
42.5
91.2
DAPI
52.3
Fig. 1c, D3
c
80.3
91.6
Ghost Dye Red 710
84.8
Fig. 1c, D7
d
27.3
89.4
Fig. 2b and Supplementary Fig. 6b
e
37.6
FSC-W
FSC-H
77.1
SSC-W
SSC-H
91.7
92.0
Fig. 3c
f
40.7
88.3
Fig. 8a

Supplementary Figure 12. Gating strategies for flow cytometry of induced and primary TECs

(a) Representative gating to isolate primary thymic epithelial cells (TEC) and non-TEC stromal cells from $CD45^{+}$-depleted human pediatric thymic stroma.

(b, c) Representative gating for day 3 (b) and day 7 (c) analysis shown in Fig. 1c.

(d) Representative gating for $mCherry^{+}$ induced TEC (iTEC) sorting for day 28 to 133 shown in Fig. 2b and Supplementary Fig. 6b.

(e) Representative gating for iTEC analysis for day 28 to 133 shown in Fig. 3c.

(f) Representative gating for $mCherry^{+}$ and $mCherry^{-}$ population sorting after iTEC/thymocyte co-culture shown in Fig. 8a.

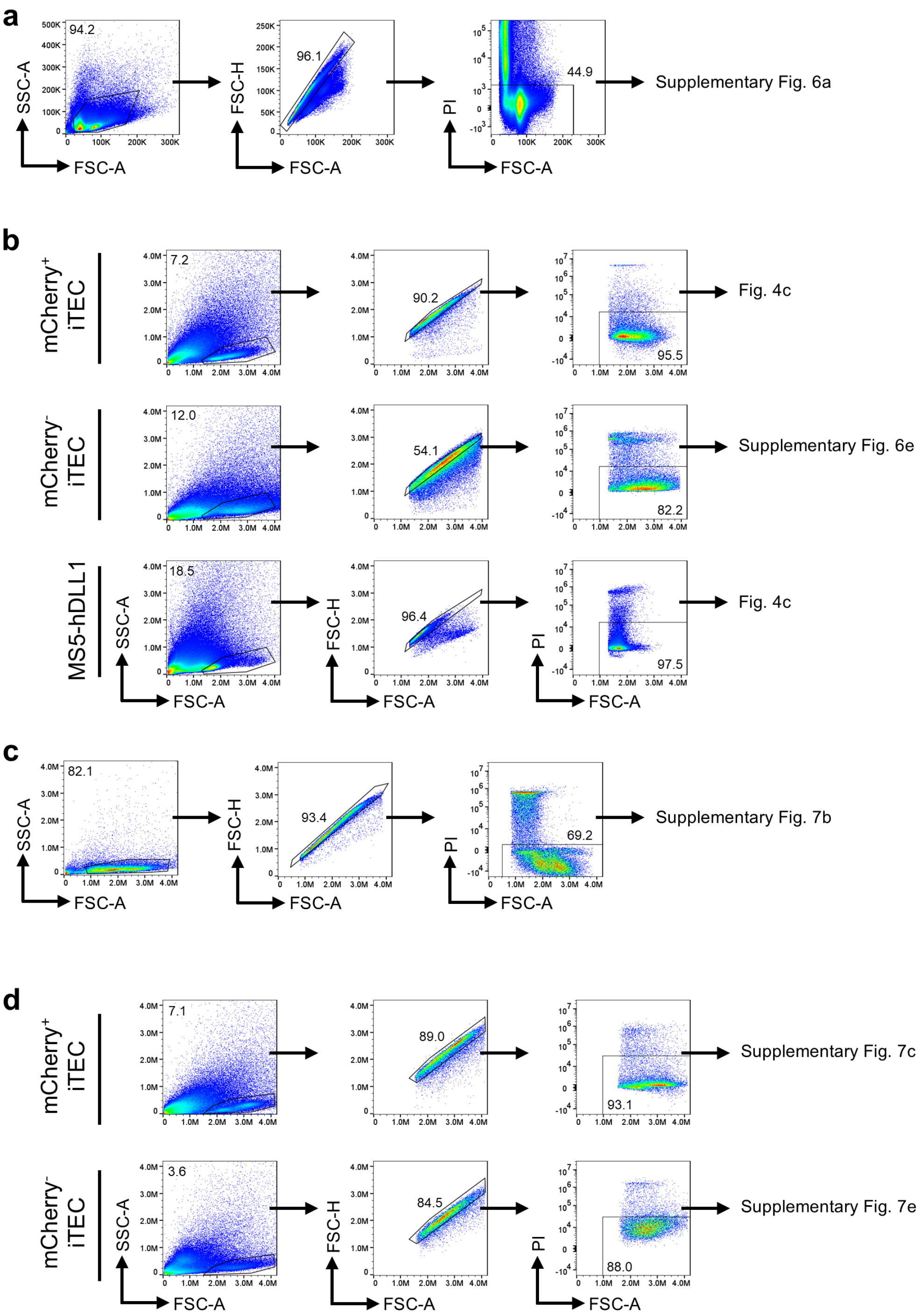
a
94.2
96.1
44.9
SSC-A
FSC-H
PI
FSC-A
Supplementary Fig. 6a
b
mCherry+ iTEC
7.2
90.2
95.5
Fig. 4c
mCherry- iTEC
12.0
54.1
82.2
Supplementary Fig. 6e
MS5-hDLL1
18.5
96.4
97.5
Fig. 4c
c
82.1
93.4
69.2
Supplementary Fig. 7b
d
mCherry+ iTEC
7.1
89.0
93.1
Supplementary Fig. 7c
mCherry- iTEC
3.6
84.5
88.0
Supplementary Fig. 7e

Supplementary Figure 13. Gating strategies for flow cytometry of thymocytes

(a) Representative gating for double-positive (DP) thymocyte sorting shown in Supplementary Fig. 6a.

(b) Representative gating for thymocyte analysis after two weeks of mCherry^{+} induced thymic epithelial cell (iTEC), mCherry^{-} iTEC, or MS5-hDLL1 co-culture with DP thymocytes shown in Fig. 4c and Supplementary Fig. 6e.

(c) Representative gating for double-negative (DN) thymocyte sorting shown in Supplementary Fig. 7b.

(d) Representative gating for thymocyte analysis after 4 weeks of mCherry^{+} or mCherry^{-} iTEC co-culture with DN thymocytes shown in Supplementary Fig. 7c, e.
