# Supplementary material for: An iPSC-based in vitro model recapitulates human thymic epithelial development and multi-lineage specification
Source: Nat Commun. 2025 Aug 25;16:7680. doi: 10.1038/s41467-025-62523-1 (PMC12378236; doi:10.1038/s41467-025-62523-1)
Supplement: Supplementary file 2 — Description of Additional Supplementary Files [file 41467_2025_62523_MOESM2_ESM.pdf]

Description of Additional Supplementary Files

Supplementary Data 1. Sources and identifiers of reagents.

Supplementary Data 2. Sequences of oligonucleotides.

Supplementary Data 3. Details of antibodies used in this study.

Supplementary Video 1. 3D-like nodules in induced TEC culture.
Z-stack displays of two 3D-like nodules in live mCherry$^+$ colonies on day 133 of induction. Scale bars, 100 μm.

Supplementary Video 2. Localization of cTEC- and mTEC-like cells in 3D-like nodules.
Z-stack displays of four 3D-like nodules in day 133 induced cells stained for PSMB11 (green), KRT5 (magenta), and DAPI (blue). Scale bars, 100 μm.

Supplementary Video 3. Localization of mesenchyme-like cells in 3D-like nodules.
Z-stack displays of four 3D-like nodules in day 133 induced cells stained for VIM (green), mCherry (magenta), and DAPI (blue). Scale bars, 100 μm.
