# Supplementary material for: An iPSC-based in vitro model recapitulates human thymic epithelial development and multi-lineage specification
Source: Nat Commun. 2025 Aug 25;16:7680. doi: 10.1038/s41467-025-62523-1 (PMC12378236; doi:10.1038/s41467-025-62523-1)
Supplement: Supplementary file 9 — Reporting Summary [file 41467_2025_62523_MOESM9_ESM.pdf]

nature portfolio

Corresponding author(s): Yoko Hamazaki

Last updated by author(s): Jul 7, 2025

Reporting Summary

Nature Portfolio wishes to improve the reproducibility of the work that we publish. This form provides structure for consistency and transparency in reporting. For further information on Nature Portfolio policies, see our Editorial Policies and the Editorial Policy Checklist.

Statistics

For all statistical analyses, confirm that the following items are present in the figure legend, table legend, main text, or Methods section.

n/a	Confirmed	
☐	☒	The exact sample size (*n*) for each experimental group/condition, given as a discrete number and unit of measurement
☐	☒	A statement on whether measurements were taken from distinct samples or whether the same sample was measured repeatedly
☐	☒	The statistical test(s) used AND whether they are one- or two-sided<br>*Only common tests should be described solely by name; describe more complex techniques in the Methods section.*
☒	☐	A description of all covariates tested
☐	☒	A description of any assumptions or corrections, such as tests of normality and adjustment for multiple comparisons
☐	☒	A full description of the statistical parameters including central tendency (e.g. means) or other basic estimates (e.g. regression coefficient) AND variation (e.g. standard deviation) or associated estimates of uncertainty (e.g. confidence intervals)
☐	☒	For null hypothesis testing, the test statistic (e.g. *F*, *t*, *r*) with confidence intervals, effect sizes, degrees of freedom and *P* value noted<br>*Give P values as exact values whenever suitable.*
☒	☐	For Bayesian analysis, information on the choice of priors and Markov chain Monte Carlo settings
☒	☐	For hierarchical and complex designs, identification of the appropriate level for tests and full reporting of outcomes
☒	☐	Estimates of effect sizes (e.g. Cohen's *d*, Pearson's *r*), indicating how they were calculated

Our web collection on statistics for biologists contains articles on many of the points above.

Software and code

Policy information about availability of computer code

Data collection	BD FACSDiva Software v9.0.1<br>SpectroFlo v3.0.3<br>QuantStudio 12K Flex Software v1.6<br>Keyence BZ-X800 analysis software v1.1.30.19
Data analysis	FlowJo v10.10.0<br>Microsoft Excel for Microsoft 365 v2402<br>GraphPad Prism v10.3.1<br>Repertoire Genesis analysis software v20180912<br>OBS Studio v30.1.2<br>Microsoft Clipchamp v3.1.10620.0<br>Cell Ranger v6.1.2<br>Scrublet v0.2.3<br>R v4.3.1<br>Seurat v4.3.0.1<br>Python v3.11.4<br>pySCENIC v0.12.1<br>Slingshot v2.10.0<br>TradeSeq v1.14.0<br>ComplexHeatmap v2.18.0<br>Velocyto v0.17.17

Scvelo v0.3.0
CellPhoneDB v5.0.0

For manuscripts utilizing custom algorithms or software that are central to the research but not yet described in published literature, software must be made available to editors and reviewers. We strongly encourage code deposition in a community repository (e.g. GitHub). See the Nature Portfolio guidelines for submitting code & software for further information.

Data

Policy information about availability of data

All manuscripts must include a data availability statement. This statement should provide the following information, where applicable:

- Accession codes, unique identifiers, or web links for publicly available datasets
- A description of any restrictions on data availability
- For clinical datasets or third party data, please ensure that the statement adheres to our policy

- Source data are provided with this paper.
- scRNA-seq data of induced and primary TECs were deposited into the GEO database under the accession number GSE275981 and are publicly available at the following URL: https://www.ncbi.nlm.nih.gov/geo/query/acc.cgi?acc=GSE275981
- RNA-seq data of TCR repertoire analysis were deposited into the GEO database under the accession number GSE275815 and are publicly available at the following URL: https://www.ncbi.nlm.nih.gov/geo/query/acc.cgi?acc=GSE275815

Research involving human participants, their data, or biological material

Policy information about studies with human participants or human data. See also policy information about sex, gender (identity/presentation), and sexual orientation and race, ethnicity and racism.

Reporting on sex and gender	Sex and gender were not considered in the study design. Sex was defined as the sex assigned at birth. Disaggregated sex and gender information was not collected. Both male and female samples were included for all analyses of two or more donors, but due to their low number, the influence of sex on the results of the study cannot be determined.
Reporting on race, ethnicity, or other socially relevant groupings	Race, ethnicity, or other socially relevant grouping information was not collected.
Population characteristics	All human research participants were pediatric patients of cardiovascular defects requiring thymus excision. Of the total 23 participants, 12 were female and 11 were male. The age ranged between 1 month and 4 years, with a median of 4 months.
Recruitment	All participants were pediatric patients of cardiovascular defects requiring thymus excision at Kyoto University Hospital for whom written informed consent was provided by the parents. Patients with chromosomal abnormalities were excluded. No compensation was provided to participants.
Ethics oversight	This study was approved by the Ethics Committee of Kyoto University Graduate School, Faculty of Medicine, and Kyoto University Hospital.

Note that full information on the approval of the study protocol must also be provided in the manuscript.

Field-specific reporting

Please select the one below that is the best fit for your research. If you are not sure, read the appropriate sections before making your selection.

☒ Life sciences ☐ Behavioural & social sciences ☐ Ecological, evolutionary & environmental sciences

For a reference copy of the document with all sections, see nature.com/documents/nr-reporting-summary-flat.pdf

Life sciences study design

All studies must disclose on these points even when the disclosure is negative.

Sample size	No statistical methods were used to predetermine sample size. Similar sample sizes (n=3-9) to that of previous studies in this field were used.
Data exclusions	No data were excluded.
Replication	A total of 3 different iPSC lines were used to demonstrate the robustness of the protocol. The number of independent experiments for replication is shown in the figure legends. All mRNA and protein expression data is based on n=3-9 replications. All immunofluorescence images are representative of n=3 or greater replications. RNA-seq and scRNA-seq data represent n=1-2 replications. scRNA-seq data showed highly similar cell population distributions on induction days 38 and 81 and were therefore not repeated for all other days.
Randomization	For each sample in all experiments, 2-12 wells in 24 well plates or 2-10 organoids in 12 well plates were randomly collected and pooled.
Blinding	No blinding for data collection or analysis was done since all methods of analysis are unbiased and unaffected by blinding.

Reporting for specific materials, systems and methods

We require information from authors about some types of materials, experimental systems and methods used in many studies. Here, indicate whether each material, system or method listed is relevant to your study. If you are not sure if a list item applies to your research, read the appropriate section before selecting a response.

Materials & experimental systems

n/a	Involved in the study
☐	☒ Antibodies
☐	☒ Eukaryotic cell lines
☒	☐ Palaeontology and archaeology
☒	☐ Animals and other organisms
☒	☐ Clinical data
☒	☐ Dual use research of concern
☒	☐ Plants

Methods

n/a	Involved in the study
☒	☐ ChIP-seq
☐	☒ Flow cytometry
☒	☐ MRI-based neuroimaging

Antibodies

Antibodies used

Antibodies are listed below in the order of Antibody, Source, Identifier, Clone, Lot, Dilution, Application.
CD45, PE/Cy7 BioLegend Cat#304016 HI30 B329567 1:50 Flow cytometry
CD235ab, PE/Cy7 BioLegend Cat#306620 HIR2 B371893 1:50 Flow cytometry
CD31, AF647 BioLegend Cat#303112 WM59 B279321 1:50 Flow cytometry
EPCAM, BV421 BioLegend Cat#324220 9C4 B305263 1:50 Flow cytometry
CD8α, BV421 BioLegend Cat#301036 RPA-T8 B440810 1:10 Flow cytometry
CD4, PE/Cy7 BioLegend Cat#300512 RPA-T4 B400204 1:10 Flow cytometry
CD45, AF700 BioLegend Cat#368514 2D1 B339566 1:10 Flow cytometry
CD3, FITC BioLegend Cat#300306 HIT3a B161348 1:10 Flow cytometry
CD45RA, AF700 BioLegend Cat#304120 HI100 B396630 1:10 Flow cytometry
TCRαβ, APC/Fire810 BioLegend Cat#306749 IP26 B445792 1:10 Flow cytometry
CD62L, BV605 BD Biosciences Cat#562720 DREG-56 3180065 1:10 Flow cytometry
TCRγδ, APC BioLegend Cat#331211 B1 B418488 1:10 Flow cytometry
CCR7, BV480 BD Biosciences Cat#566170 3D12 4200819 1:10 Flow cytometry
CD45, BUV395 BD Biosciences Cat#563791 HI30 4274479 1:10 Flow cytometry
CD8β, PE BD Biosciences Cat#641057 2ST8.5H7 4085468 1:10 Flow cytometry
CD69, PE BioLegend Cat#310906 FN50 B429659 1:10 Flow cytometry
CD19, FITC BD Biosciences Cat#555412 HIB19 1334928 1:10 Flow cytometry
CD11c, FITC BioLegend Cat#301604 3.9 B383976 1:10 Flow cytometry
CD14, AF488 BioLegend Cat#301811 M5E2 B376654 1:10 Flow cytometry
CD5, APC BioLegend Cat#300611 UCHT2 B401332 1:10 Flow cytometry
CD7, PE BioLegend Cat#343106 CD7-6B7 B363908 1:10 Flow cytometry
CXCR4, APC BioLegend Cat#306510 12G5 B316524 1:50 Flow cytometry
FOXA2, PE Miltenyi Biotec Cat#130-107-826 REA506 5201103318 1:10 Flow cytometry
SOX2, AF647 BioLegend Cat#656108 14A6A34 B296059 1:100 Flow cytometry
CD205, PE BioLegend Cat#342204 HD30 B370295 1:10 Flow cytometry
HLA-DR, PE/Cy7 BioLegend Cat#307616 L243 B443704 1:10 Flow cytometry
CD90, AF647 BioLegend Cat#328116 5E10 B309203 1:10 Flow cytometry
HLA-DQ, PE BioLegend Cat#318106 HLADQ1 B319026 1:10 Flow cytometry
CD205, AF647 BioLegend Cat#342206 HD30 B283766 1:10 Flow cytometry
HLA-A,B,C, PE/Cy7 BioLegend Cat#311429 W6/32 B372530 1:10 Flow cytometry
EPCAM, AF700 BioLegend Cat#324244 9C4 B342569 1:10 Flow cytometry
Mouse IgG2b, k isotype control, BV421 BioLegend Cat#400341 MPC-11 B351228 1:50 Flow cytometry
Mouse IgG2a, k isotype control, APC BioLegend Cat#400219 MOPC-173 B328094 1:50 Flow cytometry
Human IgG1, REA control antibody (I), PE Miltenyi Biotec Cat#130-118-347 REA293 5220206230 1:50 Flow cytometry
Mouse IgG1, k isotype control, AF647 BioLegend Cat#400135 MOPC-21 B287198 1:100 Flow cytometry
Mouse IgG1, k isotype control, PE BioLegend Cat#400114 MOPC-21 B379941 1:10 Flow cytometry
Mouse IgG2a, k isotype control, PE/Cy7 BioLegend Cat#400253 MOPC-173 B322374 1:10 Flow cytometry
Mouse IgG1, k isotype control, AF647 Novus Biologicals Cat#NBP1-43319AF647 P3.6.2.8.1 D126608 1:10 Flow cytometry
Mouse IgG2b, k isotype control, AF700 Novus Biologicals Cat#NBP1-43317AF700 MG2b D126607 1:10 Flow cytometry
EPCAM BioLegend Cat#324202 9C4 B353480 1:300 (D18), 1:600 (D28-D133) Immunofluorescence staining
TBX1 Abcam Cat#ab18530 Polyclonal GR3319454-2 1:300 Immunofluorescence staining
HOXA3 Novus Biologicals Cat#NBP1-83234 Polyclonal 000033555 1:300 Immunofluorescence staining
mCherry Abcam Cat#ab205402 Polyclonal 1026024-1 1:1500 Immunofluorescence staining
HLA-DR BioLegend Cat#307602 L243 B331589 1:600 Immunofluorescence staining
KRT5 BioLegend Cat#905504 Poly19055 B288849 1:600 Immunofluorescence staining
KRT5 Proteintech Cat#66727-1-Ig 1A1C5 10028186 1:150 Immunofluorescence staining
KRT8 Sigma-Aldrich Cat#MABT329 TROMA-1 3885482 1:600 Immunofluorescence staining
CLDN4 Thermo Fisher Cat#36-4800 Polyclonal 1073181A 1:150 Immunofluorescence staining
PSMB11 Proteintech Cat#55143-1-AP Polyclonal 09000315 1:600 Immunofluorescence staining

	Ki-67 BioLegend Cat#350501 Ki-67 B399127 1:600 Immunofluorescence staining<br>VIM BioLegend Cat#677801 O91D3 B383653 1:600 Immunofluorescence staining<br>Chicken IgY, AF488 Thermo Fisher Cat#A11039 Polyclonal 2941307 1:500 Immunofluorescence staining<br>Chicken IgY, AF594 Thermo Fisher Cat#A11042 Polyclonal 2489100 1:500 Immunofluorescence staining<br>Mouse IgG, AF488 Thermo Fisher Cat#A21202 Polyclonal 2428531 1:500 Immunofluorescence staining<br>Mouse IgG, AF647 Thermo Fisher Cat#A31571 Polyclonal 2555690 1:500 Immunofluorescence staining<br>Rat IgG, AF488 Thermo Fisher Cat#A21208 Polyclonal 2063330 1:500 Immunofluorescence staining<br>Rabbit IgG, AF488 Thermo Fisher Cat#A21206 Polyclonal 2156521 1:500 Immunofluorescence staining
Validation	All antibodies were validated by the manufacturer for the given species and application. Validation statements can be found on the manufacturers' website and data sheets for each antibody. All antibodies were used according to the manufacturers' instructions.

Eukaryotic cell lines

Policy information about cell lines and Sex and Gender in Research

Cell line source(s)	201B7: human, female, RRID:CVCL_A324, kindly gifted by Dr. Masato Nakagawa (Kyoto University)<br>409B2: human, female, RRID:CVCL_K092, kindly gifted by Dr. Keisuke Okita (Kyoto University)<br>1383D6: human, male, RRID:CVCL_UP39, kindly gifted by Dr. Masato Nakagawa (Kyoto University)<br>MS5-hDLL1: mouse, male, RRID:CVCL_VR88, purchased from Sigma-Aldrich (Cat#SCC167)
Authentication	201B7, 409B2, and 1383D6 were authenticated by STR analysis at the time of deposition to the RIKEN BioResource Research Center, and their FOXN1-mCherry reporters were authenticated by karyotyping by Chromocenter (Yonago, Japan) within five passages of their establishment. MS5-hDLL1 was verified by the vendor to be of mouse origin by a Contamination Clear panel by Charles River Animal Diagnostic Services.
Mycoplasma contamination	All cell lines were routinely tested for mycoplasma contamination and were negative.
Commonly misidentified lines<br>(See ICLAC register)	No commonly misidentified lines were used.

Plants

Seed stocks	*Report on the source of all seed stocks or other plant material used. If applicable, state the seed stock centre and catalogue number. If plant specimens were collected from the field, describe the collection location, date and sampling procedures.*
Novel plant genotypes	*Describe the methods by which all novel plant genotypes were produced. This includes those generated by transgenic approaches, gene editing, chemical/radiation-based mutagenesis and hybridization. For transgenic lines, describe the transformation method, the number of independent lines analyzed and the generation upon which experiments were performed. For gene-edited lines, describe the editor used, the endogenous sequence targeted for editing, the targeting guide RNA sequence (if applicable) and how the editor was applied.*
Authentication	*Describe any authentication procedures for each seed stock used or novel genotype generated. Describe any experiments used to assess the effect of a mutation and, where applicable, how potential secondary effects (e.g. second site T-DNA insertions, mosiacism, off-target gene editing) were examined.*

Flow Cytometry

Plots

Confirm that:

☒ The axis labels state the marker and fluorochrome used (e.g. CD4-FITC).

☒ The axis scales are clearly visible. Include numbers along axes only for bottom left plot of group (a 'group' is an analysis of identical markers).

☒ All plots are contour plots with outliers or pseudocolor plots.

☒ A numerical value for number of cells or percentage (with statistics) is provided.

Methodology

Sample preparation	Human iPSC-derived cells were dissociated with Accutase or Accumax for 7-30 min at 37 °C. For intracellular staining, cells were fixed using Fixation/Permeabilization solution (Thermo Fisher) for 30 min at room temperature. Human thymus pieces were digested for 60 to 90 min in a solution containing 100 µg/ml Liberase TM (Roche) and 200 µg/ml DNase I (Worthington) at 37 °C, after which stromal cells were enriched through magnetic depletion of CD45+ cells using MojoSort Human CD45 Nanobeads (BioLegend). Primary thymocytes were isolated from human thymi through vortexing and collecting the supernatant cells. For iTEC/thymocyte or MS5-hDLL1/thymocyte organoid analysis, the organoids were homogenized using pestles to isolate thymocytes or dissociated with Accumax for 15 min at 37 °C to isolate iTECs. After these steps, cells were washed and stained with the respective antibodies.
Instrument	BD FACSAria II (BD Biosciences), BD FACSymphony S6 (BD Biosciences), Cytek NL-3000 (Cytek Biosciences), and Cytek Aurora (Cytek Biosciences)

Software	BD FACSDiva Software, SpectroFlo, and FlowJo
Cell population abundance	10,000 events or more were collected for each analyzed sample. 5,000-100,000 cells per population were sorted for downstream qPCR and scRNA-seq analyses. The post-sorting purity of DN and DP thymocytes was determined by an immediate flow cytometry re-analysis of the sorted cells.
Gating strategy	FSC-SSC gating was specified to include all relevant cell populations while excluding debris. This was followed by doublet and dead cell removal steps. For induced cells, stained populations were gated using isotype controls for every experiment. For FOXN1-mCherry reporter lines, mCherry+ cells were gated using the parent lines as negative controls. For primary cells, stained populations were gated using unstained controls.

☒ Tick this box to confirm that a figure exemplifying the gating strategy is provided in the Supplementary Information.
